# Supplementary material for: Are carbon emissions trading and green financial instruments synergistic? -Comprehensive quantitative research based on content analysis
Source: PLoS One. 2024 Mar 7;19(3):e0298601. doi: 10.1371/journal.pone.0298601 (PMC10919601; doi:10.1371/journal.pone.0298601)
Supplement: S1 Data — (ZIP) [file pone.0298601.s001.zip › Data/地级市Z_F工作报告.zip!/┴╔─■/╠·┴δ╩╨/└┤╫╘╣1⁄2╓┌║┼í╛┬φ┐╦╩2╛▌═°í┐_2004.docx]

各位代表：

现在，我代表市第四届人民政府向大会报告工作，请予审议，并请市政协委员和其他列席会议的同志提出意见。

一、过去五年及2003年工作回顾

市四届人大一次会议以来的五年，是铁岭更加充满生机和活力，进入发展最快时期的五年，是继往开来、奋进崛起的五年。五年来，全市各族人民高举邓小平理论伟大旗帜，认真实践"三个代表"重要思想，牢牢把握加快发展这一主题，开拓进取，真抓实干，圆满完成了市四届人大历次会议确定的各项任务，国民经济和社会事业取得了显著成就。

过去的五年，是综合经济实力显著增强,人民生活水平有很大提高的五年。2003年，全市国内生产总值实现176.5亿元，比上年增长15.6%，比1998年增长62.7%，年均增长10.2%。五年累计完成固定资产投资211.7亿元，比上五年增长94.8%。社会消费品零售额实现72.5亿元，比上年增长12.8 %，比1998年增长49.2%。地区财政收入实现20.5亿元，比上年增长21.1%，比1998年增长61.9%，其中地方一般预算收入完成12.8亿元，比1998年增长86.9%。实现各类税收17.9亿元，比1998年增长51.6%。金融各项存款余额209亿元，各项贷款余额205亿元，分别比1998年增长67.1%和28.4%。经济实力的增强，带来了人民总体生活水平的明显提高，2003年，城镇居民人均可支配收入5607元，农民人均纯收入3150元，分别比1998年增长49.3%和21.2%。

过去的五年，是招商引资取得丰硕成果，对外开放实现重大突破的五年。五年共引进域外投资项目4018个，实际到位资金212.9亿元，其中投资超亿元的项目57个，域外投资已拓展到农业、工业、商贸流通、城市基础设施等各个领域，一大批知名企业集团落户我市。引进国家投资和世行贷款3.6亿元，建成了9个高标准的国家粮食储备库。新批外商投资企业172家，是上五年的1.1倍，合同外资额5.4亿美元，实际利用外资2.9亿美元，分别比上五年增长3.3倍和6.4倍，我市利用外资总额在全省已上升到第七位。累计完成外贸出口2亿美元，外派劳务5770人次，分别比上五年增长84%和1.7倍。开发区建设取得长足进展，对外开放的吸引力显著增强，已成为全市新型工业化的示范区。对外友好往来不断扩大，引智工作取得明显成效。对外开放和招商引资无论从广度、深度还是从规模上，都实现了历史性的突破。

过去的五年，是社会保障体系不断完善，就业再就业工作取得明显成效的五年。国有企业下岗职工基本生活保障向失业保险并轨取得突破性进展，累计完成并轨12万人，偿债率达86.1%。城镇企业职工基本养老保险制度日趋完善，个人帐户基金基本记清做实，参保人数达19万人，参保的离退休人员养老金100%实现了社会化发放。城镇职工基本医疗保险覆盖面不断扩大，参保人员已达23.5万人，覆盖面达72%，多层次的医疗保障体系基本建立。失业保险实现了市级统筹，确保了符合条件的失业人员按时领取失业金。城市最低生活保障制度基本建立，实现了应保尽保、动态管理。社会征缴、财政补助、企业自筹三位一体的社保资金筹措机制已经建立并逐步完善。城镇登记失业人员和最低生活保障对象全部纳入了社区管理。累计筹集扶贫帮困款物7989万元，建成了高标准的光荣院和社会福利院，新建、改建高标准中心敬老院15所，为城乡特困户建房3514户。累计安置下岗失业人员13万人次，我市引导下岗失业人员向第一产业转移、建立健全再就业服务体系等做法得到国家有关部门和省委、省政府的充分肯定。

过去的五年，是基础设施建设进展最快，城乡面貌发生巨大变化的五年。进一步完善城市总体规划，实施大规模的基础设施建设和环境综合整治，新建和改造主次干道36条、小街小巷115条，城市道路框架基本拉开。建成了驻跸园音乐广场、城南热源厂、引铁法煤层气等20多项重大基础设施工程，城市功能更加完善。市区绿化覆盖率达到38.7%，比1998年提高2.2个百分点，美化、亮化、净化水平上了一个新台阶。改造旧城区76万平方米，改造治理住宅小区31个。邮政、通信网络规模不断扩大，综合业务处理能力和技术水平明显提高。五年新增建制镇3个，市级以上中心镇达到12个。完成了南城子水库除险加固、东辽河防洪等5项重大农田水利基础设施工程，西丰诚信水库开工建设。五年投资21.9亿元，新增公路里程478公里，新建、改建黑色路面2214公里，是上五年的2.9倍，提前1年实现了乡乡通油路的目标，通油路的行政村达到970个，占总数的63.7%，被省政府授予"公路建设先进单位"，连续4年被评为省公路建设文明市。国土资源管理和环境保护工作得到加强，耕地开发复垦及整治取得明显成效，"一控一达标"工作被省政府授予一等奖。

过去的五年，是各项社会事业日益繁荣，民主法制和精神文明建设不断开创新局面的五年。科技工作取得新成果，8个农作物新品种通过省级鉴定，推广应用10项先进农业生产技术，建成了市农业高新技术园并正式投入生产，市农科院被批准为国家大豆改良中心铁岭分中心，高新技术企业发展到7家，有4项科研成果获省科技进步二等奖。教育改革与发展稳步推进，实现了农村公办教师基本工资县统筹，我市首创的旨在提高农村小学教育教学质量的"春晓工程"在全省推广。教育布局更趋合理，办学条件得到明显改善。高中招生规模不断扩大，高等教育、职业教育、民办教育快速发展。文化事业日益繁荣，成功地举办了铁岭首届国际民间艺术节，我市被中国曲艺家协会命名为"曲艺小品之乡"。全市共有297件文艺作品获省以上奖励，电视剧《今天是个好日子》获全国"五个一工程"奖，《刘老根》第二部在全国热播，极大地提高了铁岭的知名度。卫生事业迈出新步伐，农村卫生院、所建设得到加强，卫生监督与疾病控制体制改革取得重要进展，社区卫生服务的覆盖面进一步扩大，医疗质量管理水平不断提高。计划生育"三为主"方针得到切实落实，进一步巩固了低生育水平。群众体育蓬勃发展，全民健身月活动被国家体育总局评为优秀组织奖，竞技体育捷报频传，我市输送的运动员有4人6次获世界冠军，9人次两平一破世界纪录，有8人入选2004年雅典奥运会国家集训队。广播电视基础设施和设备得到改善，节目更加丰富多彩，广播电视人口覆盖面进一步扩大。民主法制和精神文明建设得到切实加强，共办理人大代表建议、政协提案2320件，满意率达95%以上。"三五"普法和依法治市工作取得很大成效，人民调节网络更加健全，法律服务领域不断拓展，法律援助制度走上正规化，社会治安秩序保持稳定。全面推行了行政执法责任制和评议考核制，强化了政府法制监督和执法人员的培训工作，认真、公正地开展行政复议应诉工作，规范了行政执法行为，维护了相对人的合法权益。广泛开展多种形式的精神文明创建活动，我市城乡共建经验在全国推广，有3家单位被命名为全国精神文明建设先进单位。强化科技拥军，积极为驻铁部队解难题、办实事，驻铁部队广大官兵对地方经济和社会发展给予了大力支持，我市首次被评为省级"双拥模范城"。

各位代表，刚刚过去的2003年，是我市全面建设小康社会和实现"三个新突破"的开局之年。面对突如其来的"非典"疫情，全市上下万众一心，众志成城，严格落实防治"非典"的各项措施，坚持以经济建设为中心不动摇，取得了防治"非典"和经济建设的双胜利。一是招商引资实现新突破。共引进域外投资项目355个，实际到位资金50亿元，其中超亿元的大项目41个。新批外商投资企业36家，合同外资额2.3亿美元，外商实际缴资额1亿美元，分别增长87.7%和46%。外贸出口实现5600万美元，增长21.2%。外派劳务1700人，增长20.6%。二是固定资产投资创历史新高。完成固定资产投资60亿元，增长37.4%。三是农村经济结构调整和农业产业化取得新进展。粮食总产量25.3亿公斤，为5年来最好水平，粮食与非粮作物实际种植面积比例调整到60:40。新建村外畜牧小区60个，完成植树造林39.5万亩、7954万株。农村二、三产业实现总产值235亿元，增长16%，实现利润14.1亿元，实缴税金2.7亿元，分别增长14.6%和17.4%。四是工业经济呈现速度和效益同步快速增长的新局面。规模以上工业完成工业总产值126.6亿元，增长28.8%。实现工业增加值45亿元，增长23.3%。实现利税11.5亿元，增长7.5%。启动建设工业园区42个，摆进项目106个。完成技改投资10亿元，开发新产品135项，实现新产品产值7.3亿元。五是民营经济有新发展。全市民营经济发展到11.9万户，从业人员42.5万人，实现增加值63亿元，增长18.4 %。六是社保和再就业工作取得新成效。社保试点各项工作顺利完成，在财力紧张的情况下投资1500万元建成了目前省内规模最大、功能齐全的市级劳动力市场，启动了放心早餐工程，开展了"腾岗位、献爱心"活动，全年开发公益性岗位4328个。七是城乡建设迈出新步伐。市区新建、改建12条主次干道和13条街巷路，植树42.2万株，新增公共绿地6.6公顷。建成了岭东排水暗渠，银北热源厂投入使用，柴河风光带一期整治河道工程全面完成。房地产开发42万平方米，治理改造居民小区17个，解决社区办公用房20处，是力度最大的一年。新建、改建黑色路面1011公里，首次突破一千公里。

审计、技术监督、统计、物价、侨务、地震、档案、药品监督、供销、烟草专卖、规划、人防、气象、农机、安全、残联、消防、地方志、老龄等工作也都取得了新成绩。

随着2003年各项任务的完成，本届政府走过了五年不平凡的历程。回顾五年来的工作，我们深切地感到，这些成就的取得，得益于我们在工作中始终做到了五个坚持：

1、始终坚持以对外开放和招商引资为中心。五年来，我们不断强化对外开放和招商引资的中心地位，坚持不懈地加强软硬环境建设，使对外开放和招商引资真正成为全市上下的共识和自觉行动，实现了历史性的突破，对全市经济和社会发展起到了巨大的拉动作用。可以说，没有对外开放和招商引资，就没有铁岭五年来的快速发展。更为可喜的是，对外开放和招商引资极大地推进了全市思想解放的进程，使广大干部群众的思想观念发生了深刻变化，市场经济意识和开放意识明显增强，为铁岭经济跨越式发展奠定了坚实的思想基础。

2、始终坚持以结构调整为主线。五年来，我们按照"调整优化第一产业，突出加强第二产业，大力发展第三产业"的总体思路，不断加大经济结构战略性调整和改组的力度。农业和农村经济结构发生了历史性变化，形成了"一带、五区、六大基地"的种植业新格局，畜牧业和林业已成为农业经济的两大主导产业，城乡二元经济结构正在逐步改变。工业经济结构调整初见成效，新型建材、机械、轻工、能源、农副产品加工初现主导产业端倪。从1999年起，全市规模以上工业企业一举摘掉了长达8年整体亏损的帽子，开始走上速度和效益同步快速增长的良性循环轨道。特别是"工业立市"思想的提出，在全市上下引起强烈反响并形成广泛共识，为实现"三个新突破"和全面建设铁岭小康社会找到了成功的路径。

3、始终坚持以改革为动力。五年来，我们坚持以改革总揽全局，加快推进以转制退出和股份制改造为重点的国有企业改革，以稳定和完善家庭承包责任制为重点的农村改革，以裁减冗员、理顺财务管理为重点的粮食流通体制改革，以国库集中支付、部门预算、政府采购等为重点的财政改革，以城市经营性用地招标拍卖为重点的国土资源使用制度改革，以公房出售、住房分配货币化为重点的住房制度改革以及科技、教育、文化、卫生等各项改革，在一定程度上消除了影响发展的体制性、机制性障碍，为全市经济和社会发展注入了新的活力，提供了强大动力。

4、始终坚持以经济和社会协调发展为目标。五年来，我们始终坚持物质文明、政治文明、精神文明一起抓，深入实施"科教兴市"战略，不断加大对科技教育的投入，制定了鼓励高新技术产业发展、吸引人才等优惠政策，科技教育事业获得了长足发展，人才总量进一步增加。切实加强文化、卫生、体育、广播电视等各项社会事业和精神文明建设，扎扎实实地推进文化名城创建工作，使经济和社会更加协调发展，"红学文化之乡"、"小品艺术之乡"、"体育冠军之乡"、"煤电能源之城"四张城市名片进一步在海内外叫响。

5、始终坚持以执政为民为根本宗旨。五年来，我们切实加强政府自身建设，认真落实廉洁自律的各项规定，大力纠正部门和行业不正之风，严肃查处各种违法违纪案件；完成了市县两级机构改革和合乡并镇，进行了两轮行政审批制度改革，推行了"一站式"办公服务；严厉打击各种违法犯罪活动，妥善处理民族宗教事务，认真受理人民群众来信来访和市民投诉，我市被授予全国信访工作优秀集体。所有这些，都是为了保持政府同人民群众的血肉联系，建设精干高效的服务型政府，为人民群众创造舒适安全的生活环境。归根到底，是为了忠实地实践全心全意为人民服务的根本宗旨，做到立党为公，执政为民。

各位代表，五年来各项成就的取得，是省委、省政府和市委正确领导的结果，是市人大、市政协监督与大力支持的结果，是全市上下团结一心、共同奋斗的结果。在此，我代表市人民政府，向辛勤工作在各条战线上的全市各族人民，向热情支持政府工作的人大代表、政协委员，向各民主党派、工商联、无党派人士及社会各界人士，向解放军驻铁部队指战员和武警官兵、中省直驻铁单位，向广大域外投资客商，向所有关心支持铁岭改革开放和现代化建设的海内外朋友表示衷心的感谢并致以崇高的敬意！

各位代表，越是形势比预料的好，越要保持清醒的头脑，越要看到经济和社会发展中存在的困难和问题。当前，我们在前进中面临的主要矛盾和问题是：经济发展虽然速度很快，但总量还比较小，有些经济指标在全省还比较靠后，实现强市富民目标，任重道远，压力很大；经济结构不尽合理，运行质量不高，经营方式还比较粗放；"三农"问题仍很突出，农民增收难度加大；就业再就业任务繁重，社保资金严重不足，部分群众生活还比较困难；对外开放环境还需进一步改善，有的部门和工作人员以经济建设为中心的思想树得不牢，服务意识不强，办事效率还不高；官僚主义、形式主义、铺张浪费、腐败现象和行业不正之风还一定程度地存在；安全生产形势不容乐观，特别是发生了昌图12·30特大事故，给人民群众的生命财产造成重大损失。这些矛盾和问题必须引起我们高度重视，并在今后的工作中认真加以解决。

二、今后五年的指导思想和奋斗目标

今后五年，正逢国家振兴东北老工业基地和省委、省政府加快辽西北开发的重要战略机遇期，同时面临国际产业转移、国内南资北移的大好时机；我市经济正处于发展最快的历史时期，显示了强劲的后发优势；广大干部群众求发展、谋振兴的氛围十分浓厚，全市上下政通人和，精神振奋，这些都为我们加快发展提供了极为有利的条件。只要我们牢固树立机遇意识、发展意识、创新意识、责任意识，利用一切可以利用的有利条件，调动一切可以调动的积极因素，励精图治，真抓实干，就一定会取得新突破，实现大发展，在振兴老工业基地中大有作为，向党和人民递交一份无愧于先人、无憾于来者的满意答卷。

政府工作的指导思想是：以邓小平理论和"三个代表"重要思想为指导，深入贯彻十六大和十六届三中全会精神，坚持五个统筹，抢抓四大机遇，以加快发展为主题，以项目招商为中心，以强市富民为目标，牢固树立工业立市思想，以发展民营经济为主，加速工业化，推进农业产业化，提高城镇化水平，全力推进经济结构调整，重点培育"五大主导产业"，加快建设 "三大基地"，深入实施科教兴市、人才强市战略，坚持全面、协调、可持续发展观，切实加强物质文明、政治文明、精神文明建设，为建设经济发达、文化繁荣、生活富裕、环境优美的新铁岭而奋斗。

今后五年的主要奋斗目标是：

1、做大经济总量，实现强市升位。国内生产总值年均增长18.3 %以上，到2008年，GDP总量达到407亿元。地区财政收入达到44.8亿元，年均增长16.9 %。五年完成固定资产投资536亿元，年均增长20%。社会消费品零售额实现154亿元，年均增长16.4 %，力争主要经济指标在全省位次前移。进一步完善社会保障体系，大力改善创业和就业环境，努力增加城乡居民收入。到2008年，城镇居民人均可支配收入和农民人均纯收入分别达到8240元和4420元，年均增长8 %和7 %，人民生活水平再上一个大台阶。

2、突出抓好大项目招商，实现全方位、宽领域的对外开放新格局。在更大范围、更宽领域、更高层次上参与国内国际竞争，构筑一流的对外开放环境，基本构建起不受地域限制，没有制度壁垒，各种生产要素自由流动、合理配置的开放型发展模式。坚持引进项目与引进资金、智力、技术相结合，不断拓展对外开放新领域。特别要依托资源优势，突出抓好大项目招商，力争每年引进投资超亿元的大项目20个以上，五年引进域外资金300亿元，实际利用外资10亿美元。优化出口商品结构，建成肉鸡、生猪、花生、参茸四大出口基地，培植服装、蔬菜、肉鸡等八大主导出口商品，五年完成外贸出口7亿美元，年均增长30%。

3、全力加快工业经济发展，实现由传统农业地区向工业城市的跨越。主动承接沈阳产业转移，大力调整经济发展战略布局，全面开发、改造、建设铁西工业区，搞好铁岭县县城搬迁的规划论证，力争早日启动，建成50个市县乡三级工业园区，使其成为铁岭经济发展新的增长点。做大做强华晨、精工等33户年产值超亿元的企业, 培育铁煤集团、铁岭电厂、陆平等5个年产值超20亿元的企业集团，新建一批具有较强竞争力的企业，形成机械加工制造、汽车零部件配套加工、轻工、高新技术产业和现代服务贸易"五大主导产业"，建成能源工业、优质安全农副产品生产加工、新型建筑材料工业"三大基地"。大力发展生物技术、新材料、机电一体化等高新技术产业，重点扶持北亚药用油、北辰彩板等28户高新技术企业，加快用高新技术改造传统产业，基本实现产业优化升级。到2008年，工业总产值达到700亿元，其中规模以上工业总产值达到382亿元，三次产业结构比由目前的29：37：34调整到18：47：35，初步实现由传统农业地区向工业城市的跨越。

4、加速推进农业产业化，实现解决"三农"问题的新突破。强化农业基础地位，用工业理念研究和解决农业问题，实现城乡经济社会协调发展。大力发展农村二三产业，加快农村剩余劳动力转移。新上和扩建龙泉乳业、万顺达淀粉等投资1000万元以上的产业化龙头企业50个，重点培育奶牛、玉米、生猪等20个产业化链条，以此为牵动，加强优质、安全农产品基地建设，加快推行农产品市场准入制度。培育各具特色的县域主导产业，发展壮大县域经济，使我市由农业资源大市向农产品加工大市转变，由传统农业向现代农业转变，初步解决农业效益低、农民增收缓慢等突出矛盾和问题。

5、大力发展民营经济，实现以民营经济为主体的新型所有制结构。营造鼓励全民创业的浓厚氛围，不断完善并认真落实促进民营经济发展的各项政策措施，清除一切不利于民营经济发展的条条框框，为民营经济发展营造最优的政策环境、服务环境和法制环境，让一切创造社会财富的源泉在市场经济大潮中充分涌流。实施龙头、规模、品牌、园区"四大驱动战略"，形成骨干崛起、结构优化、素质提高、特色鲜明的民营经济发展新格局。到2008年，民营经济在国民经济中的比重达到70%以上。

6、加快推进各项改革，实现经济体制和管理机制的全面创新。国有中小企业资本全部退出国有，对启动无望的企业依法实施破产。全面推进以土地流转制度、农村税费改革和社会化服务体系建设为重点的农村改革，深化国有资产管理、财税、科教文卫和行政管理等各项改革，最大限度地消除影响生产力发展的体制性、机制性障碍，基本建立起较为完善的现代产权制度、符合社会主义市场经济要求的经济体制和便捷高效的行政管理机制。

7、加大城乡基础设施建设力度，实现城镇化水平和环境质量的全面提升。继续坚持"上档次、塑形象、大突破"的方针，进一步完善城市总体规划和专项规划，推进中心城市向东、向北发展，初步形成北方山水城市格局。加大经营城市力度，加快城乡基础设施建设，全面完成城区道路和管网改造，形成完善的基础设施体系。建设柴河风光带、城市垃圾处理厂等30项重点工程，开展大规模、高水准的绿化美化亮化，全面提升城市生态和生活环境质量，塑造城市新形象。创新小城镇建设和管理体制，通过放宽户籍政策、鼓励多元化投入等措施，引导农村人流、物流向小城镇集中，加快农村城镇化进程，到2008年，城镇化水平提高到45%。

8、扎扎实实地开展文化名城建设工作，实现经济与社会、人与自然的协调发展。构建先进的文化创新体系，大力发展文化产业，围绕四张城市名片，加快文化设施建设，培育具有鲜明地域特色的文化品牌，开展具有广泛影响的大型文化活动，做好市志编纂工作，挖掘历史文化资源，把我市建成真正的文化名城。加快发展各项社会事业，整合高等教育资源，力争建成一所市属本科大学，加强职业技术教育及培训基地建设，形成比较完善的国民教育体系、科技创新体系、全民健身体系和医疗卫生体系。深入开展依法治市和形式多样的精神文明创建活动，全面提高人民群众的科学文化素质、思想道德素质和健康素质。高度重视人才工作，认真落实吸引人才的各项优惠政策，继续实施"一村一名大学生"计划，五年引进大中专毕业生3000人，特别要引进一批高层次人才，为各类人才在铁岭施展才华创造优良的外部环境。切实加强计划生育、环境保护和资源管理工作，使经济发展与资源环境相协调。

各位代表，上述奋斗目标是在我市全面建设小康社会方案和实现"三个新突破"实施意见的基础上提出来的，实现这些目标，难度是不言而喻的。但收获总在耕耘后，成功从来历艰辛。我们已经具备了跨越式发展的基础和条件，只要我们万众一心，埋头苦干，我们的目标可以实现，我们的目标一定能够实现！

三、2004年主要工作安排

2004年是新一届政府任期的第一年。做好今年的工作，具有十分重要的意义。为此，要突出工作重点，狠抓关键环节，特别要开展好"安全年、项目年、环境年"活动，为全市经济发展和社会稳定创造一个良好的开端。

今年全市经济和社会发展的主要预期目标是：国内生产总值实现207亿元，增长17％。全社会固定资产投资完成72亿元，增长20％。地区财政收入21.5亿元，比上年预算增长16％，其中地方一般预算收入9.9亿元（新体制口径），比上年预算增长17.7%。社会消费品零售额83亿元，增长14.8％。城镇居民人均可支配收入6056元，农民人均纯收入3370元，分别增长8%和7%。安置再就业4万人。人口自然增长率控制在3.31‰以下。

为实现上述目标，重点抓好九项工作：

（一）努力提高对外开放水平，力争再引进一批对全市经济发展起到重大拉动作用的大项目

创新招商方式，着力抓好以商招商、以情招商，组织开展好赴韩国、新加坡、香港、广州等10次大型招商活动，力争引进投资超亿元的大项目20个，全年招商引资实际到位资金50亿元，实际利用外资1.2亿美元。继续实行领导包大项目责任制，全力抓好玉米秸秆造纸、50万吨酒精等18个已签约和在谈项目的跟踪落实，促其早建设、早投产。按照科学论证、致力创新、突出特色的原则，切实加强项目源建设，规划、包装好30个重点项目。全力扩大外贸出口，全年完成出口8000万美元，增长43％。加大工作力度，年内完成出入境检验检疫局建设。加强对外经济技术交流与合作，外派劳务2000人以上。以推行项目代办制、并联审批、规范费用减免为重点，大力优化对外开放环境，努力营造"亲商、安商、养商、富商"的良好氛围。加快完善开发区帽山、桑园两个起步区的基础设施，提升开发区的整体形象，充分发挥对外开放的窗口和龙头作用。

（二）加快工业调整与改造步伐，塑造铁岭工业的新形象

抢抓国家振兴东北老工业基地的历史机遇，抓好清河电厂"以大代小"、铁岭电厂二期、铁煤集团技术改造等35个项目的规划，搞好与国家和省有关部门的对接，争取更多的项目进入国家和省的笼子。全面启动铁西工业区改造，重新焕发铁西工业区的活力，加快改善铁西的生产生活环境。按照统一规划、合理布局、完善功能、突出特色的原则，加快县乡工业园区建设，力争年内每个园区都摆进一批项目，使园区建设初具规模。继续深化国企改革，今年国有中小工业企业转制退出40户。下力气抓好企业技术创新，全年完成技改投资10亿元，开发新产品150种。大力发展高新技术产业，实施高新技术产业化项目12项，力争再有2户企业进入省级高新技术企业，新发展民营科技企业60户。全市规模以上工业完成工业总产值157亿元，实现增加值56亿元，实现利税14.5亿元，分别增长24 %、24%和26%，继续保持速度和效益同步快速增长。

（三）加速推进农业和农村经济结构调整，进一步提高农业产业化水平

突出抓好"一带、五区、六大基地"建设，不断扩大优质、安全、高效农产品种植面积。以村外畜牧小区建设、人工牧草种植、奶牛生产为重点，提高畜牧业生产规模化、集约化、专业化、市场化水平，奶牛饲养量发展到3万头，新建村外畜牧小区50个，畜牧业产值实现48亿元。完成植树造林35万亩，植树6000万株。无公害和绿色食品基地面积达到100万亩，获无公害食品认证50个。重点扶持一批农业产业化龙头企业，建设和完善146个农业产业化基地。加强农业基础设施建设，完成水源工程500项，综合机械化水平达到39％以上，加快改善农业生产条件。加强农业技术培训，提高农民的科技素质。

（四）营造鼓励创业、扶持创业的良好环境，促进民营经济和第三产业快发展大发展

重点扶持一批超亿元的民营企业，使其迅速做强做大。全市民营经济增加值实现75.6亿元，增长20％，实缴税金4.5亿元，增长21.6％。大力发展第三产业，完成裕路商业城建设并投入运营。积极寻找合作伙伴，开发建设辽北果菜批发市场。加快邮政、通信网络建设，着手建立辽北邮政物流中心，积极开办新兴邮政、电信业务。完成《铁岭市旅游发展总体规划》修编，围绕红学文化，规划建设几处高水准的人文景观，使其成为铁岭旅游的新亮点，旅游总收入达到5亿元以上。

（五）继续加大社保和再就业工作力度，妥善安排困难群体的基本生活

偿还并轨人员债务90%以上。巩固和扩大养老保险成果，积极探索农村养老保险新路子，依法加大基金筹集与管理力度，确保离退休人员养老金100％社会化发放和失业金按时足额发放。抓好医疗保险扩面工作，覆盖率达到75％。加快社保网络和街道、社区劳动保障平台建设，将社保和就业工作全部纳入社区管理。认真落实再就业优惠政策，加强职业培训基地建设，全面启动下岗失业人员再就业小额贷款业务，继续开展"腾岗位、献爱心"就业援助活动，千方百计增加就业岗位。完善城市居民最低生活保障制度，巩固应保尽保成果。建设8所区域性中心敬老院。坚持救助式扶贫与开发式扶贫相结合，深入开展扶贫帮困活动，妥善安排好困难群体的生产生活。

（六）实施一批重大基础设施和公益设施建设项目，全面改善城市人居环境

加大经营城市特别是土地招标拍卖工作力度，力争筹集资金1亿元以上。改建、扩建文化街、南马路、柴河街北段、市区北出口等6条主要街路。搞好柴河风光带一期工程和龙首山旅游景区的开发建设，市区植树20万株，新建南柳、北市、汇工街三个游园，新增公共绿地5公顷。以铁西、岭东、城南为重点，开发住宅面积65万平方米，旧城区改造面积26万平方米，完成4个居民小区的环境改造，全部完成55个社区办公用房建设。搞好乡镇规划编制，加快12个市级以上中心镇建设，深入开展市容环境和村镇环境的综合整治，提高城乡人居环境的整体水平。加快农村公路网建设，完成300公里村通油路工程，全面完成102线北段二级路改建。加大环境监管和投入力度，基本建成开原、调兵山城市污水处理厂，开工建设铁岭市城市垃圾处理厂，争取建设铁岭污水处理厂中水回用工程和污泥综合利用工程，使我市环境质量有一个较大改善。

（七）积极稳妥地实施财税体制改革，加大金融对经济发展的支持力度

完善预算编制改革，实施部门综合预算。扩大财政国库集中支付范围，规范财政支出行为。积极稳妥地推进农村税费改革，进一步减轻农民负担。继续深化政府采购、非税收入"收支两条线"管理、县区经费财政统管等各项财政改革，不断增强政府财政调控和保障能力。全面推行税费征管体制改革，坚持依法治税，强化税收征管，确保各项收入按新体制规定及时足额入库。优化财政支出结构，大力压缩一般性支出，保证公教人员工资、社保资金和各项法定支出。调整信贷结构，改善金融服务，加大对高新技术产业、绿色农业和民营企业的支持力度。强化金融监管，整治社会信用环境，建设金融安全区。加强审计工作，突出搞好财政预算执行、重点建设项目、重点行业的审计监督，推进领导干部经济责任审计，严肃财经纪律，提高资金使用效益。

（八）大力发展各项社会事业，精心组织好第二届国际民间艺术节

推广科技成果20项，完成专利申请350项，科技成果鉴定30项，引进新品种、新技术40项。进一步落实"以县为主"的教育管理体制，重点抓好控辍保学，通过扶贫助学专项资金和"寒窗基金"资助特困学生5000名。加大教育投入，改造危房8万平方米，对53所农村中小学布局进行调整。全面推进基础教育课程改革，全面提高教育教学质量。制定扶持政策，促进文化事业和文化产业发展。组织参加好省第六届艺术节，修复银冈书院，开工建设市图书馆，将其建成铁岭标志性建筑。整合卫生资源，加强疾病预防控制体系、卫生监督执法体系和医疗救治体系建设，完善突发公共卫生事件的应急处理机制。继续实施"光明行动"，让更多的白内障患者重见光明。加强农村医疗保健工作，改善农民的就医条件。深入开展计划生育优质服务，不断提高整体工作水平。继续实施全民健身计划，构建全民健身多元化服务体系。加强电视传输网络建设，完成广电大厦主体工程，丰富广播电视节目。广泛开展形式多样的精神文明创建活动，争创"省级文明城市"。落实优抚和安置政策，巩固和发展"双拥"成果，增进军政、军民团结。继续按照"政府主办、社会参与、市场运作"的原则，精心举办好第二届国际民间艺术节，弘扬铁岭民间艺术，进一步提高我市在海内外的知名度。

（九）切实加强民主法制建设，创造稳定的社会环境

认真执行人大及其常委会的各项决议、决定，自觉接受人大的依法监督和政协的民主监督，努力提高人大代表建议和政协提案办理质量。加强同民主党派、工商联、无党派人士的联系，发挥工会、共青团、妇联等群团组织的作用。继续推进政务、村务公开，加强基层民主政治建设。认真落实"四五"普法规划，实施"民主法制示范村创建"和"用法律武装农民工程"，提高全社会的法律素质和法制化管理水平。完善法律援助制度，保护困难群体的合法权益。积极推进行政执法体制改革，继续抓好行政执法人员培训和资格认证工作，全面提高行政执法人员的素质。认真清理行政许可规定和许可主体，规范行政许可行为。依法履行行政复议职责，维护管理相对人的合法权益，提高依法行政水平。严格落实归口办理、分级负责的信访责任制，认真解决群众反映的实际问题，减少越级访和重复访。继续开展严打整治活动，搞好公安管理体制改革，加快社会治安防控体系建设，提高处理群体性突发事件的能力，创造稳定的社会环境。认真吸取昌图12·30特大事故血的教训，提高全民的安全意识，强化经常性的安全检查，加大安全隐患的整治力度，杜绝重特大安全事故的再度发生。深入开展民族乡镇"手拉手"帮扶活动，促进民族地区经济发展。全面贯彻党的民族宗教信仰自由政策，依法管理宗教事务，积极引导宗教与社会主义社会相适应。

四、建设人民满意的政府，为实现经济和社会事业快速发展提供坚强保证

各位代表，本次大会将选举产生新一届市政府领导班子。新一届政府肩负着光荣而艰巨的历史使命。建设让人民满意的政府，是全市人民的期望，更是实现各项奋斗目标的保证。为此，新一届政府必须按照勤政、廉洁、务实、高效、创新的要求，切实加强和改进政府工作，塑造充满希望的政府形象。

亲民为民，时刻保持同人民群众的血肉联系。各级领导干部和广大公务员要经常想一想生活还比较困难的群众，经常想一想自己过去的艰苦生活，时刻不要忘本。要怀着对人民群众的深厚感情去工作，关心城乡困难群众的生活，了解他们的需求，千方百计地为群众排忧解难，真正做到立党为公，执政为民。不断拓宽政府与市民的沟通渠道，各级政府要充分发挥市民投诉中心的作用，建立领导与群众定期对话制度，面对面地倾听群众的意见、建议和要求。各级政府在做出涉及千家万户利益的重大决策前，要履行听证程序，认真听取和吸纳群众的意见。要继续开通行风热线，自觉接受人民群众的监督。加强政府同人民群众的联系，着力营造聚精会神搞建设、一心一意谋发展的良好氛围，增强广大干部群众抢抓机遇，加快发展的紧迫感、责任感和使命感，引导好、保护好、发挥好他们投身全面建设小康社会的积极性、主动性和创造性。

不务虚名，踏踏实实地为老百姓办实事。要树立正确的政绩观，坚决克服形式主义和官僚主义，不搞花架子，不做表面文章，不搞不切实际的形象工程，把群众的希望和要求作为政府工作的第一信号，把群众满意作为评价政府工作的第一标准。各级领导要从不必要的迎来送往和繁琐事务中解脱出来，腾出时间和精力，深入实际调查研究，围绕百姓最希望解决的问题，多办得人心、暖人心的好事、实事，把党和政府的温暖送到群众的心坎上。要把抓落实作为改进作风的重要内容，问责到人，做到事事有人抓、有人管、有人督办，确保各项工作落到实处。要建立健全奖惩责任制，对求真务实、埋头苦干的干部进行褒奖，对好大喜功、弄虚作假的干部进行惩戒，在政府机关中形成勤政为民、踏实苦干的浓厚风气。

创新机制，努力提高办事效率。加快转变政府职能，进一步简政放权，把那些应由市场调节、法律裁决、中介组织解决的事项从政府职能中分离出去。狠抓机关的作风建设，努力改进服务，提倡和推行专题办公、现场办公，方便基层和群众。继续搞好机构改革、行政审批制度改革和事业单位改革，进一步削减审批事项，简化审批程序。对需多个部门审批的事项，推行联合审批、定期会签制度，在审批事项较多的部门推行一个窗口进、一个窗口出的"窗口式办文"制度，提高办事效率和行政效能。加快电子政务建设，积极创造条件实行网上审批。强化"一站式"办公服务中心职能，逐步把全部审批事项纳入到中心审批。下力气打造诚信铁岭，加快建立与国际接轨的透明高效的服务机制，建设手续简、收费低、服务优、办事快的一流的经济发展环境。

加强学习，切实提高广大公务员的素质。在政府机关中大力倡导认真学习的风气、研究问题的风气。市政府将聘请专家学者，定期举办知识讲座，帮助研究论证一个时期的重大问题。开展创建学习型机关活动，加强对广大公务员市场经济知识、法律知识、业务知识的培训，不断汲取新知，努力提升自身素质，克服本领恐慌，增强工作的前瞻性。坚持学以致用，务求学习效果，紧密结合思想和工作实际，拓宽新思路，采取新举措，不断提高广大公务员特别是各级领导干部科学判断形势的能力、驾驭市场经济的能力、应对复杂局面的能力、依法行政的能力和总揽全局的能力。要在政府机关中引入竞争机制，实行优胜劣汰，保持公务员队伍的生机和活力。

廉洁从政，不辜负人民群众的厚望和重托。铁岭目前经济还不发达，相当一部分群众生活比较困难。广大公务员特别是各级领导干部，一定要牢记 "一粥一饭，当思来之不易；半丝半缕，恒念物力维艰"。要大力发扬艰苦奋斗、勤俭节约的优良传统，坚决制止和反对大手大脚、铺张浪费的行为，与人民群众同甘共苦，风雨同舟。要加强对政府各级领导干部的教育和监督，切实落实廉洁自律各项规定，做到严于律己，廉洁从政。认真清理乱收费、乱摊派、乱罚款、乱拉赞助，大力纠正部门和行业不正之风，让人民群众到政府机关办事心情舒畅。要加大对违法违纪案件的查办力度，对干扰经济建设、妨碍招商引资、侵害群众合法权益的行为，要严肃处理，绝不姑息迁就，以反腐败的实际成果取信于民，努力建设人民满意的政府。

各位代表！我们已经踏上了全面建设铁岭小康社会的伟大征程。让我们乘着十六大的东风，高举邓小平理论伟大旗帜，认真实践"三个代表"重要思想，紧密团结在以胡锦涛同志为总书记的党中央周围，在市委的坚强领导下，勇敢地担负起振兴铁岭经济的历史重任，不辱使命，不负重托，扎实工作，奋发进取，共同创造铁岭美好的未来！
